# Integrated ONT Full-Length Transcriptome and Metabolism Reveal the Mechanism Affecting Ovulation in Muscovy Duck (*Cairina moschata*)

**DOI:** 10.3389/fvets.2022.890979

**Published:** 2022-07-08

**Authors:** Junyuan Lin, Liyan Ge, Xiang Mei, Yurui Niu, Chu Chen, Shuisheng Hou, Xiaolin Liu

**Affiliations:** ^1^College of Animal Science and Technology, Northwest A&F University, Xianyang, China; ^2^Ministry of Agriculture Key Laboratory of Animal Genetics Breeding and Reproduction (Poultry), Institute of Animal Science, Chinese Academy of Agricultural Sciences, Beijing, China

**Keywords:** ovulation, Muscovy duck, full-length transcriptome, metabolomics, lipid metabolism

## Abstract

Ovulation is a complicated physiological process that is regulated by a multitude of different pathways. In comparison to mammalian studies, there are few reports of ovulation in Muscovy ducks, and the molecular mechanism of ovarian development remained unclear. In order to identify candidate genes and metabolites related to Muscovy duck follicular ovulation, the study combined Oxford Nanopore Technologies (ONT) full-length transcriptome and metabolomics to analyze the differences in gene expression and metabolite accumulation in the ovaries between pre-ovulation (PO) and consecutive ovulation (CO) Muscovy ducks. 83 differentially accumulated metabolites (DAMs) were identified using metabolomics analysis, 33 of which are related to lipids. Combined with data from previous transcriptomic analyses found that DEGs and DAMs were particularly enriched in processes including the regulation of glycerophospholipid metabolism pathway, arachidonic acid metabolic pathway and the steroid biosynthetic pathway. In summary, the novel potential mechanisms that affect ovulation in Muscovy ducks may be related to lipid metabolism, and the findings provide new insights into the mechanisms of ovulation in waterfowl and will contribute to a better understanding of changes in the waterfowl ovarian development regulatory network.

## Introduction

The Muscovy duck (*Cairina moschata*) is an outstanding meat breed with high protein content and superb meat quality (1), but its strong nesting behavior causes brooding behavior, which leads to low egg production and severely limits the current duck industry's development requirements. Waterfowl ovaries contain numerous follicles of various sizes and stages of development which resemble a cluster of grapes, with each follicle acting as an individual grape. A sluggish growing process occurs of the follicle development from primordial follicle development to ovulation, a strict hierarchical system and selection of dominant follicles are required (2). After developing into hierarchical follicles, they will be filled with yolk and grow quickly. At peak period of duck production, the biggest follicle from the hierarchical follicles will select to ovulate every day, which is a unique characteristic of waterfowl follicle growth and ovulation (3, 4). Only a few follicles can become dominant follicles, and the majority of them have the possibility of atresia at different phases of development (5). However, the mechanism affecting Muscovy duck ovulation is still unclear, and it might be linked to a series of complicated processes.

For more than 10 years, short-read RNA-Seq has been utilized, in which the RNA or cDNA needs to be fragmented during the sample preparation procedure, resulting in the loss of some information in the original transcript. In recent years, the technology for gene sequencing has advanced tremendously, and Oxford Nanopore Technologies (ONT) is currently at the forefront of the area (6, 7). ONT has overcome the read-length barrier to accomplish ultra-long read, single-base detection at the genome-wide level (8, 9). Metabolomics is a technique for detecting metabolites (small compounds) in blood, tissues, and other biological materials, with the presence and relative concentration of these molecules acting as can be used as evidence of processes and functions (10). The “omics” sciences of metabolomics, proteomics, transcriptomics, and genomics are closely linked and are considered the cornerstones of systems biology (11). A single omics approach provides limited insights into complex molecular pathways and complex biological events in cells and organisms. With the maturation of multi-omics analysis methods, more and more omics are being utilized together to more systematically explain the complicated correlation between phenotype and mechanism (12, 13).

In recent years, many research on waterfowl follicles genomics have been published, most of which have focused on single transcriptome data, such as Xu et al. (14) analyzed the transcriptome sequencing of pigeon ovaries at pre-ovulation, post-ovulation and 5–6 days after ovulation, Zhang et al. (15) analyzed and compared the transcriptome of ovarian tissues from chickens with relatively greater and lesser egg production, and Hu et al. (16) studied the dynamics of the transcriptome of goose ovaries during late embryonic and early post-hatching stages. However, there has been few published research on a comprehensive comparative metabolome–transcriptome analysis of waterfowl follicles. We explored the changes in the development of female Muscovy ducks from metabolites and gene expression, as well as the potential underlying mechanisms affecting ovulation in Muscovy ducks, using metabolomics combined with transcriptomics analysis. It is hoped that this research will contribute to increasing the theoretical guidance of Muscovy duck genetic breeding and improving the actual breeding efficiency.

## Materials and Methods

### Sample Preparation and Extraction

Muscovy duck was achieved from Shaanxi Anda Agricultural Development Co., Ltd (Shannxi, China). The ovaries of 12 Muscovy ducks were sampled, including six ovaries from pre-ovulated (PO, 22 weeks) and six ovaries from consecutive-ovulated (CO, 40 weeks) Muscovy duck. Each set of ducks had a similar body weight and had the same genetic background and appearance. They were also grown under the same feeding management conditions. All methods and procedures were conducted in accordance with the relevant guidelines formulated by the Ministry of Agriculture of the People's Republic of China and involved animal manipulation was approved by the Faculty Animal Care and Use Committee of Northwest A&F University (Shannxi, China). All samples were immediately frozen in liquid nitrogen and transferred to −80°C for further use. After the samples were thawed on ice, weigh the sample 50 mg, add 1,000 μl of pre-cooled extractant (70% aqueous methanol solution containing 1 μg/ml of 2-chlorophenylalanine as internal standard), add pre-cooled steel beads, homogenize for 3 min at 30 Hz, remove the beads, vortex for 1 min, let stand on ice for 15 min, centrifuge for 10 min at 4°C, 12,000 *r*/min, remove the supernatant into the injection vial liner tube. The supernatant was taken into the liner tube of the injection vial and used for LC-MS/MS analysis, and cDNA sequencing was performed on a MinION platform (Oxford Nanopore Technologies, Oxford, UK) (17).

### HPLC Conditions and ESI-QTRAP-MS/MS Analysis

The sample extracts were analyzed using an LC-ESI-MS/MS system (UPLC, Shim-pack UFLC SHIMADZU CBM A system, https://www.shimadzu.com/; MS, QTRAP^®^ System, https://sciex.com/). The analytical conditions were as follows, UPLC: column, Waters ACQUITY UPLC HSS T3 C18 (1.8 μm, 2.1 mm ^*^ 100 mm); column temperature, 40°C; flow rate, 0.4 ml/min; injection volume, 2 μl; solvent system, water (0.04% acetic acid): acetonitrile (0.04% acetic acid); gradient program, 95:5 V/V at 0 min, 5:95 V/V at 11.0 min, 5:95 V/V at 12.0 min, 95:5 V/V at 12.1 min, 95:5 V/V at 14.0 min. LIT and triple quadrupole (QQQ) scans were acquired on a triple quadrupole-linear ion trap mass spectrometer (QTRAP), QTRAP^®^ LC-MS/MS System, equipped with an ESI Turbo Ion-Spray interface, operating in positive and negative ion mode and controlled by Analyst 1.6.3 software (Sciex). The ESI source operation parameters were as follows: source temperature 500°C; ion spray voltage (IS) 5,500 V (positive), −4,500 V (negative); ion source gas I (GSI), gas II (GSII), curtain gas (CUR) was set at 55, 60, and 25.0 psi, respectively; the collision gas (CAD) was high. Instrument tuning and mass calibration were performed with 10 and 100 μmol/L polypropylene glycol solutions in QQQ and LIT modes, respectively. A specific set of MRM transitions were monitored for each period according to the metabolites eluted within this period.

### Metabolomics Data Analysis

Principal component analysis was used to observe the degree of variability between different groups and between samples within groups. The variables with less correlation are analyzed by Partial Least Squares-Discriminant Analysis (PLS-DA). PLS-DA is a multivariate statistical analysis method that extracts independent variable X and dependent variable Y, then calculates the correlation between the components. Compared with PCA, PLS-DA can maximize the distinction between groups, which is conducive to finding different metabolites. Orthogonal Partial Least Squares Discriminant Analysis (OPLS-DA) combines orthogonal signal correction (OSC) and PLS-DA methods to screen difference variables by removing irrelevant differences. The data obtained for differentially accumulated metabolites is by the preliminary screening of differential metabolites from the VIP value (Variable Importance in Projection, VIP) of the OPLS-DA model, and then combining the *P*-value and fold change of univariate analysis to further screen out differential metabolites. Threshold of variables determined to be important in the projection (VIP) scores ≥1.0 together with fold change ≥2 or ≤ 0.5 was adopted to assess differentially accumulated metabolites. Kyoto Encyclopedia of Genes and Genomes (KEGG, http://www.genome.jp/kegg/) was utilized to search for the metabolite pathways. Furthermore, we conducted integrative studies of metabolomics with our previous transcriptomics data (18) by displaying metabolites related with genes on the pathways. Integrated analysis was performed using BMKCloud (www.biocloud.net).

### Statistical Analysis

For this study, transcriptomic and metabolomic analyses used samples from the same participants and time points. Three independent biological replicates were tested for transcriptomic analysis, and six independent biological replicates were conducted for metabolic analysis. The same sample is used for combined transcriptome and metabolome analysis.

## Results

### UPLC-MS/MS-Based Quantitative Metabolomic Analysis of Ovaries

**Figure 1:**
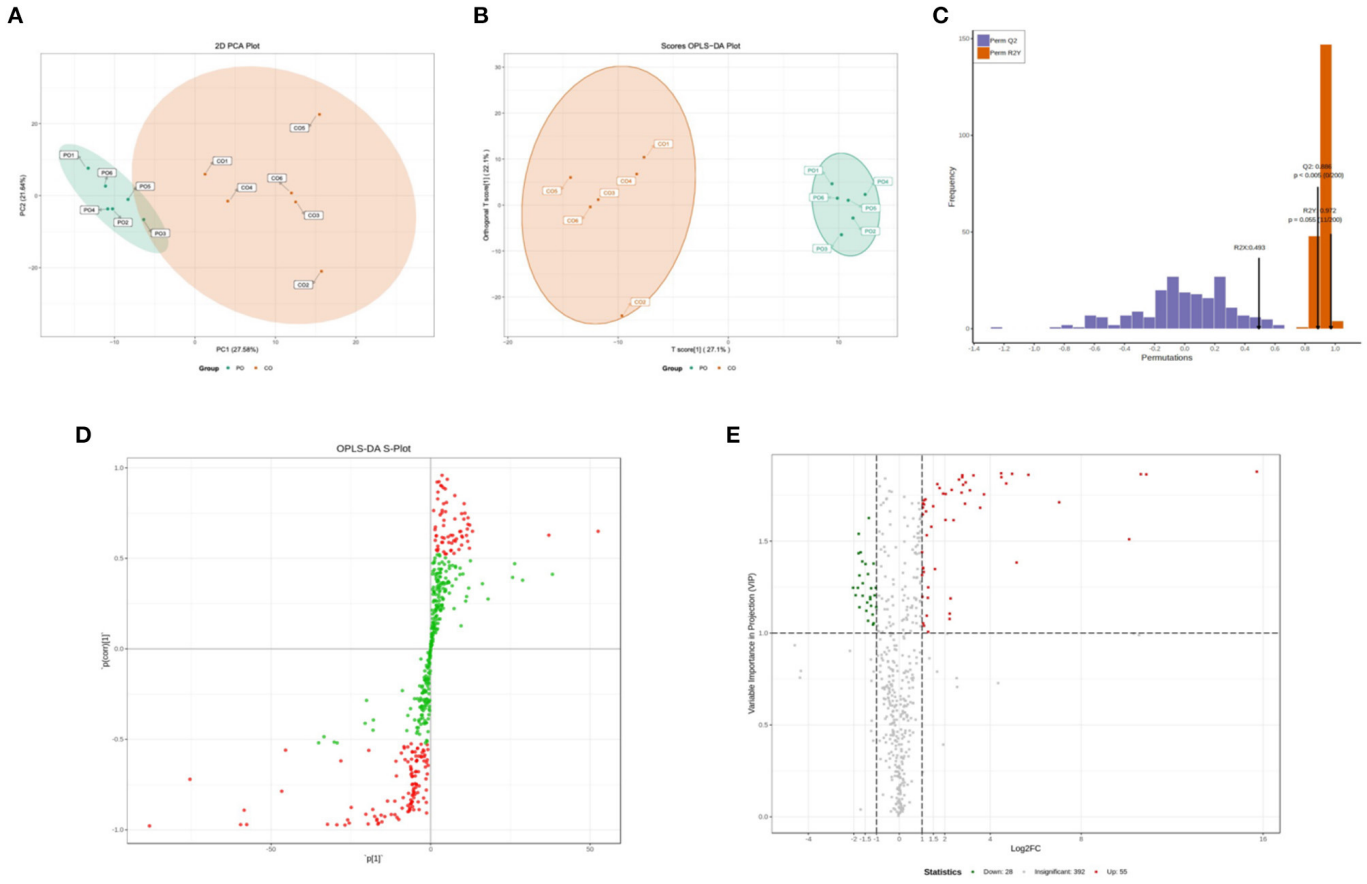
Qualitative and quantitative analysis of the metabolomics data. **(A)** Principal component analysis (PCA) of metabolomics. **(B)** Orthogonal partial least squares discriminant analysis (OPLS-DA) scores. **(C)** OPLS-DA model validation. **(D)** Variable importance in projection (VIP) values, the metabolites closer to the upper right and lower left corner indicate their more significant differences. Red dots indicate VIP values ≥1 for these metabolites, and green dots indicate VIP values <1 for these metabolites. **(E)** Volcano plots of all identified metabolites, each point in the volcano plot represents a metabolite. Green dots in the plot represent down-regulated differentially expressed metabolites, red dots represent up-regulated differentially expressed metabolites, and black represents metabolites detected but with insignificant differences. The X-axis represents the logarithmic value of the quantitative difference multiples of a metabolite in two samples; the Y-axis represents the VIP value.

To better understand the changes in the CO-PO group, the PCA and OPLS-DA score plots that we constructed using the acquired metabolomic data. The PCA and OPLS-DA modes of Muscovy Duck are shown in Figure 1. Segregation trends between groups in metabolic physiology were lightly detected using the unsupervised PCA of the entire set of measured analytes. The result showed that the two sets of samples show a good separation trend as can be seen from the PCA model (Figure 1A). To maximize the discrimination between the two groups, and thus, we performed orthogonal partial least squares discriminant analysis (OPLS-DA). The OPLS-DA model maximizes the discrimination between groups and is more conducive to the subsequent search for differential metabolites (Figure 1B). To verify this model from a statistical point of view, this model performed 200 random permutation and combination experiments on the data, the model parameters were obtained as R2X = 0.493, R2Y = 0.927, and Q2 = 0.886 (Figure 1C). According to the prediction parameters of the evaluation model, R2X, R2Y and Q2 indicated that OPLS-DA model suffered from neither excessive randomness nor overfitting, which indicated that the model was stable and reliable and had a high predictive ability. Therefore, these data could be used for subsequent analysis.

### Analysis of Differentially Abundant Metabolites

**Figure 2:**
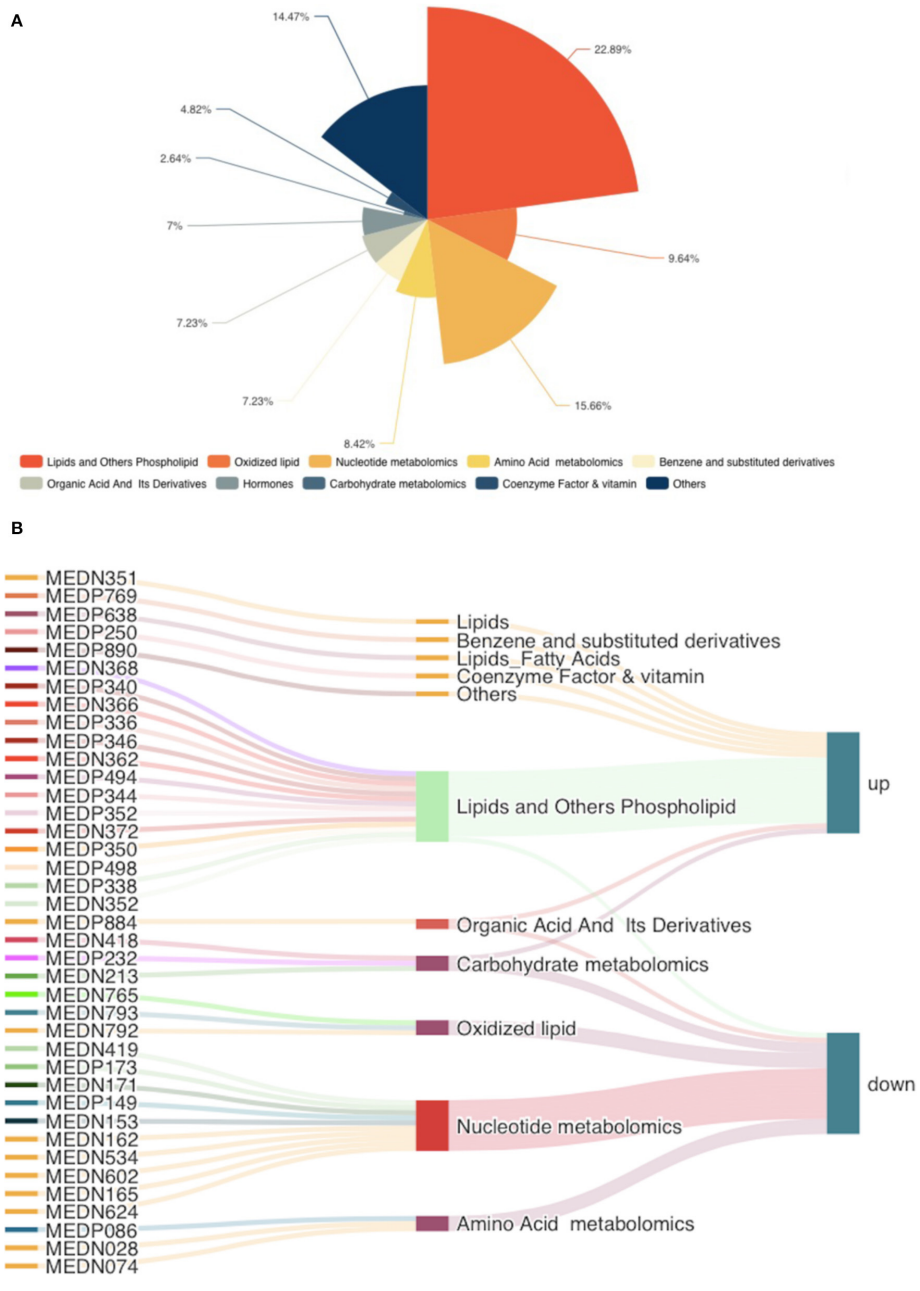
The classes of differentially accumulated metabolites. **(A)** Pie charts showing different classes of total DAMs, different colors indicate different classes. **(B)** The top 20 up-regulated and down-regulated DAMs were illustrated in the Sankey diagram.

**Figure 3:**
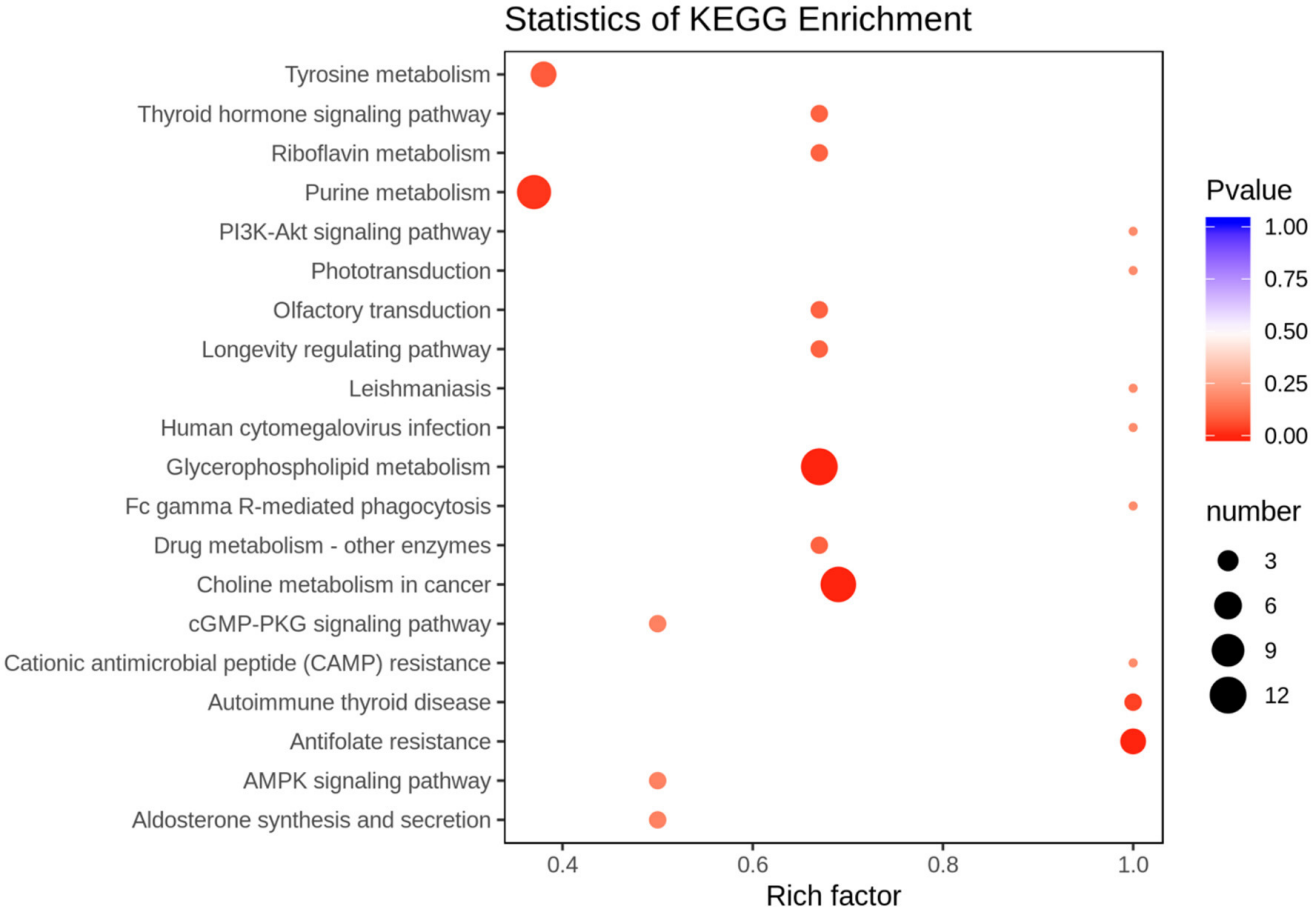
Top 20 Kyoto Encyclopedia of Genes and Genomes (KEGG) pathway terms (*P* < 0.05) enriched by differentially accumulated metabolites (DAMs). The X-axis means rich factor. The Y-axis represents the KEGG pathway terms.

According to the basis of the OPLS-DA results, the variable importance projection (VIP) of the OPLS-DA model obtained from the multivariate analysis can also be combined with the *P*-value of univariate analysis or the fold change value (fold change) to further filter different metabolites. Variable importance in projection (VIP) >1.0 together with fold change ≥2 or ≤ 0.5 was used as the screening standard for differential abundant metabolites (Figure 1D), a total of 83 metabolites showed significant differences in the PO vs. CO comparison, 55 metabolites in the PO group were significantly upregulated compared with those in the CO group. By contrast, 28 metabolites were significantly downregulated. Their corresponding information was also shown in volcano plots, which could intuitively indicate the differences in the metabolites between the PO-CO groups (Figure 1E). The DAMs in CO vs. PO could be categorized into 10 classes, the classes of Lipids (39.76%), Nucleotide metabolomics (15.66%), Amino acid metabolomics (8.42%), Benzene and substituted derivatives (7.23%) and Organic Acid and Its Derivatives (7.23%) accounted for a large proportion (Figure 2A). It is surprising that the most of top 20 up-regulated DAMs belong to the classification of Lipids Others Phospholipid (Figure 2B). The classes of Lipids and Others Phospholipid including Lysopg 18:1, Lysope 18:1, Lysope 18:0, Lysope 16:0, Lysope 14:0, Lysopa 18:0, Lysopa 16:0, Lysopc 14:0, Lysopc 16:0, Lysopc 16:1, Lysopc 18:0, Lysopc 18:1, Lysopc 18:2, Lysopc 20:1, Lysopc 20:2, Lysopc 17:0, Lysopc 15:0, Lysopc 18:3 were increased (Supplementary Table 1). Pathway analysis was subsequently conducted based on the Kyoto Encyclopedia of Genes and Genomes (KEGG) database. DAMs are mainly grouped under the terms of Glycerophospholipid metabolism, Choline metabolism in cancer, Antifolate resistance, Purine metabolism, Autoimmune thyroid disease (Figure 3).

### Integrated Analysis of Metabolomics and Transcriptomics

**Figure 4:**
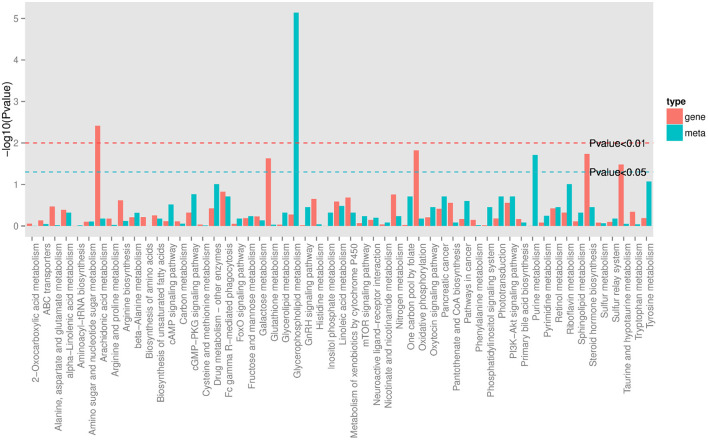
KEGG Pathway co-enriched analysis of DEGs and DAMs. Bar chart showing the number of metabolites covered by each enriched pathway. The Y-axis indicates –Log10 (*P*-value) of metabolites while the X-axis indicates the pathway name. Red: gene; blue: metabolites.

**Figure 5:**
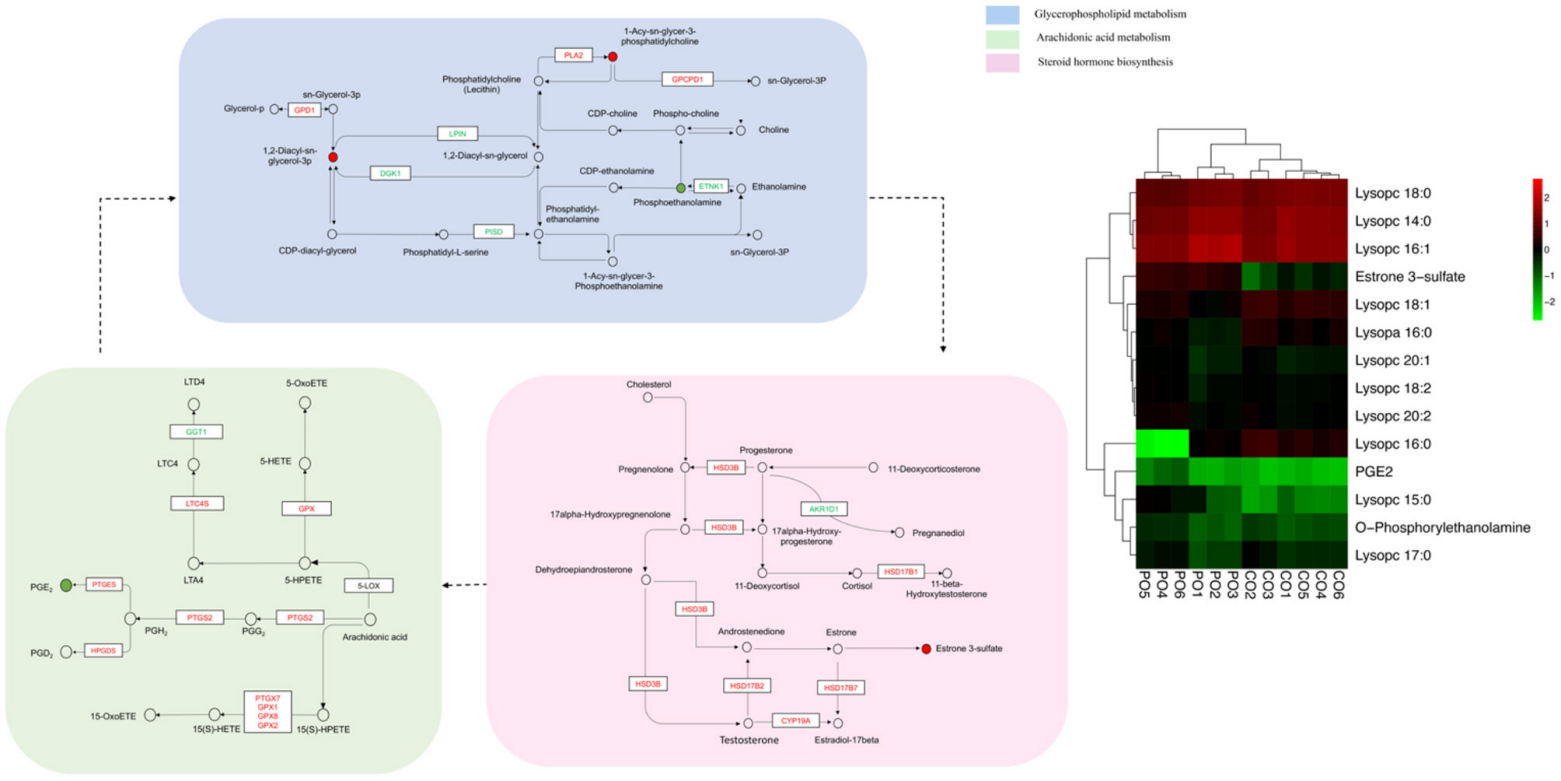
Constructed three key pathways that integrated relative metabolite contents and gene expression. Different colors were used to represent down-regulated DEGs (blue), up-regulated DEGs (red). The heatmaps are drawn according to the metabolomics data. Columns and rows in the heatmap represent groups and metabolites, respectively. The color scale indicates fold changes (Log10) in gene expression.

In order to track the change process of the metabolome more deeply, we join the transcriptome for multi-omics joint analysis. Previous transcriptomics data showed that a total of 3,046 DEGs (1,046 up-regulated and 2,000 down-regulated) were identified in the PO compared with the CO group (18). A comprehensive analysis of metabolomics and transcriptomics was performed, and 55 Co-enriched pathways were derived (Figure 4), the lipid metabolism-related pathways were dramatically changed, of which we focused on three pathways including Arachidonic Acid metabolism, Glycerophospholipid metabolism and Steroid hormone biosynthesis pathways. We present the relationship between the differential gene expression and differentially abundant metabolites associated with these pathways with pictures in the figure (Figure 5). Integrated analysis among these three altered pathways revealed that Glycerophospholipid metabolism was the top enriched pathway, with 14 DEGs and 12 DAMs, including Lysopa 16:0, Lysopc 14:0, Lysopc 16:0, Lysopc 16:1, Lysopc 18:0, Lysopc 18:1, Lysopc 18:2, Lysopc 20:1, Lysopc 20:2, Lysopc 17:0, Lysopc 15:0 and O-Phosphorylethanolamine, all these LPCs and LPAs involved in glycerophospholipid metabolism pathways were significantly increased, whereas the O-Phosphorylethanolamine was down-regulated. Through ARA and steroid hormone biosynthesis pathway analysis, PGEs and estrone 3-sulfate were found to be dramatically changed in the PO group. 12 DEGs were increased, whereas 13 DEGs were decreased, according to the transcriptional levels (Supplementary Tables 2, 3).

## Discussion

There are a complex series of changes that occur during follicular development in Muscovy ducks. Only a few follicles are chosen to develop into the dominant follicle and the majority of follicles undergo atretic degeneration before ovulation (5). Follicular development and eventual ovulation are essential to ensure egg production and promote fertility in waterfowl. Multi-Omics technologies create an encouraging environment for researchers to explore biology mechanisms of complex systems (19, 20). Here, we integrated transcriptomics and metabolomics to analyze DEGs and DAMs of ovaries, explored the correlation between gene expression changes and metabolite abundance in the enriched pathway, as well as the further elucidated potential mechanisms that may influence ovulation in Muscovy ducks. Analyzing the DAMs at different phases of ovarian follicle development revealed a considerable degree of lipid metabolism change. Lipids play a variety of roles in the body, including energy storage, biofilm formation, and signaling molecules (21, 22). Understanding the lipid metabolic pathways and the genes involved is essential for further research into lipid biological functions. To date, there have been a lot of studies focusing on the mechanisms by which mammalian lipid metabolism regulates ovulation (23–25), while very little has been reported in waterfowl studies.

Arachidonic acid pathway was one of the pathways that dramatically changed. Arachidonic acid is a polyunsaturated fatty acid (PUFA) that is converted to cyclic compounds such as prostaglandins, prostacyclins, and thromboxanes by cyclooxygenase (COX), and non-cyclic compounds by lipoxygenase (LOX) to generate HETE and leukotrienes (LT) (26, 27). According to studies, arachidonic acid levels are higher in the ovaries after ovulation than the ovaries before ovulation. Arachidonic acid influences ovulation by regulating granulosa cell-oocyte communication and oocyte maturation paracrine signals (22). Ovulation has also been linked to the activation of metabolism-related alterations in COX and LOX (28). PGG2 can be further catalyzed by PTGS2 to form prostaglandin H2 (PGH2) (29), and then under the action of prostaglandin E synthase (PGES), which is encoded by the PTGES gene, to produce PGE2. Through its surface G-protein-coupled receptor, PGE2 can function in concert with gonadotropin (hCG) signaling (30). It is worth mentioning that PTGS2 is the rate-limiting enzyme in prostaglandin E2 (PGE2) synthesis and is expressed at high levels in pre-ovulatory follicle granulosa cells, which is consistent with our transcriptome findings. According to Fujimori et al. (31), the results of the study strongly indicate that the prostaglandin E2 produced by COX-2 is involved in the ovulation of the Medaka. Zebrafish have increased PTGS2 expression during ovulation, whereas mice with deficient PTGS2 expression are unable to ovulate (32). The PTGS2/PGE2 biosynthesis pathway may at least partly regulate the induction of genes involved in the steroid biosynthesis pathway. Extensive literature has shown that the PTGS2-activated pathway is required for successful ovulation (32, 33). Indeed, both 5- LOX and 15-LOX levels have been proven to be significantly increased during pregnancy (34). GPX is a prominent antioxidant enzyme that is significantly more expressed in mature oocytes than in immature oocytes, and it can protect cells from oxidative damage during ovulation. Consistent with the literature, our research found that the transcript level of GPX1, GPX2 and GPX8 all significantly increased. Uterine contractions are induced by 5-HETE, which is produced by 5-LOX *via* GPX (35). 15-LOX generates 15-HETE through GPX, and studies have revealed that 15 (S)-HETE levels are considerably higher during ovulation in both cattle and females (36). Ovulation is actually an inflammatory reaction, and LTs have inflammatory and immune functions and play essential roles in follicle maturation, ovulation, and luteal formation. LTA4 is a precursor of LT biosynthesis that is very unstable and readily metabolized by LTA4 hydrolase to LTB4 or by LTC4 synthase to LTC4. More leukotrienes are produced in the plasma of pregnant women. LTB4 regulates the recruitment of NK and T cells (37, 38) and enhances the innate immune defense of pregnant women (39). LTC4 is significantly increased during ovulation in rats, and our study also corroborates this result.

One of the most critical processes for ovarian development and reproduction in animals is the steroid biosynthesis pathway (40, 41). In mammalian follicles, estrogen is mostly synthesized in granulosa cells, the precursor of estrogen synthesis (androgens) is provided by membrane cells for granulosa cells, and progesterone is primarily secreted by the corpus luteum after ovulation (42, 43). Whereas it is very different between waterfowl and mammals (44), the waterfowl has a unique hierarchical follicular system and does not form a corpus luteum after ovulation, and steroid hormones are synthesized and secreted by the granulosa and membranous cell layers of the follicle (45, 46). Progesterone, a steroid hormone secreted by the gonads, is produced by the granulosa cells of the pre-ovulatory follicle, and androgens and estrogens are synthesized in the inner and outer layers of the membrane cells, respectively. Starting with the formation of pregnenolone (P5) which is catalyzed by cholesterol *via* cholesterol side-chain cleavage enzyme (P450SCC/CYP11A1), followed by the conversion of pregnenolone to progesterone by 3β-hydroxysteroid dehydrogenase (3β-HSD) (42, 47). Despite the evident differences in follicular development, numerous studies have shown that the pathway catalyzing cholesterol to progesterone is basically the same as that of mammals. The other branch, the conversion from pregnenolone to DHEA and subsequently from DHEA to A4, is regulated by 3β-hydroxysteroid dehydrogenase (3βHSD) (48). A4 generates estrone which in turn generates estrone 3-sulfate, and Estrone generates E2 in response to HSD17B7. A4 can also be converted to testosterone (T) by catalysis of 17βHSD2 and eventually aromatized to estradiol (E2) by cytochrome P450 aromatase (P450arom/CYP19) (49). Here, our transcriptome analysis data enrichment analysis showed significant differences in the PO-CO group for the genes HSD-3β, HSD17b1, HSD17b2, HSD17b7, and CYP19a AKD1R1 involved in the steroid biosynthesis pathway, and metabolomic results showed a significant increase in the accumulation of progesterone and estrone as well. HSD-3β is one of the key enzymes in the steroidogenesis pathway, whose expression steadily increases in granulosa cells of growing hierarchical follicles (50, 51). Through a series of endocrine processes in the embryonic ovary, HSD-3β and gonadotropins are likely to promote the initiation of germ cell meiosis (52). HSD17B2 is an endoplasmic reticulum enzyme that inactivates hormones. The gene encoding HSD17B2 is expressed in the endometrium and placental capillaries (53–55). Aromatase is encoded by the CYP19A1 gene. CYP19A1 knockout mice are unable to synthesize estrogen, resulting in granulosa cell apoptosis and follicular atresia, indicating that CYP19A1 plays a decisive role in the normal ovulation of the ovary (56, 57). The steroid biosynthetic pathway may be involved in follicle development and eventually ovulation, and its regulatory in ducks need to be proven in further experiments.

Furthermore, Glycerophospholipid metabolism is the top enriched metabolism pathway. Glycerophospholipids are the most abundant phospholipids and serve as a source of energy for cellular metabolism. They also play an important role in cell signaling and cell membrane development. Diacylglycerol (DAG) is a glycerolipid metabolic intermediate and the world's first known lipid second messenger (23). Phosphatidylinositol (4, 5)-biphospholipids are hydrolyzed to produce DAG, and protein kinase C (PKC) is the major effector (58). Diacylglycerol kinase (DGK) cleaves diacylglycerol to form phosphatidic acid (PA), and PLA-type enzymes deacylated PA to produce LPA, which refers to 1-acyl-2-hydroxy-sn-glycero-3-phosphate and belongs to a group of lysogenic glycerolipids, although other forms exist as well (59). Metabolomic analysis showed that Lysopa 18:0 and Lysopa 16:0 were highly accumulated in the PO-CO group in our study. Lysopa 16:0 is the most abundant form of LPA in humans, and LPA has been attributed to follicular growth and ovulation in human (60), bovine (61), and porcine (62). LPA is released in the endometrium of cattle, induces the expression of COX-2, progesterone, and PGE2, and is significantly increased during human pregnancy (63). Moreover, numerous studies have suggested that LPA may plays a role in oocytes (64). In human oocytes cultured *in vitro*, the addition of lysophosphatidic acid to immature oocytes increased cell cycle protein B1 levels in MI and MII stage oocytes and boosted the maturation rate of oocytes *in vitro* considerably (62). LPA has also been proven to activate the mitogen-activated protein kinase (MAPK) pathway, which enhances oocyte nuclear maturation (62, 65). These findings suggest that ovarian-derived LPA is an essential regulator of normal ovarian development and ovulation. In addition, our metabolome profiling various types of LPC involved in glycerophospholipid metabolism were altered, with Lysopc 14:0, Lysopc 16:0, Lysopc 16:1, Lysopc 18:0, Lysopc 18:1, Lysopc 18:2, Lysopc 20:1, and Lysopc 20:2 significantly upregulated in the PO-CO group. Phospholipase A2 (PLA2) metabolizes membrane lipids to arachidonic acid (AA) and LPC. LPC is highly mobile within intact cells, acts as a cytoplasmic messenger to signal downstream processes and gene expression in the nucleus (66), and is also capable of activating multiple second messengers, and LPC in follicular fluid is involved in many essential processes in signal transduction and cell-to-cell communication. Human follicular metabolome results indicate that LPC (13:0) and LPC (18:0) are highest in oocytes and that LPC may be involved in the regulation of follicular development and oocyte maturation, which may be closely related to ovulation (67, 68). LysoPCs are a source of long-chain fatty acids. At the start of egg-laying, Muscovy ducks may generate inflammation in local tissues such as the reproductive tract. As a substrate for macrophage membrane remodeling, lysoPCs assist macrophages to invade tissues rapidly during inflammation. LysoPCs are tightly linked with ovulation (69).

In conclusion, we identified underlying metabolomics differences among pre-ovulation and consecutive ovulation Muscovy ducks, conducted the integrated analysis with transcriptomics. The results suggested that Arachidonic Acid metabolism, Glycerophospholipid metabolism, and Steroid hormone biosynthesis pathways may be the key processes associated with the mechanism of Muscovy duck ovulation. Candidate genes, metabolites and pathways identified in this study require further experiments to demonstrate. In addition, this finding, combined with mammalian systems, contributes to a more comprehensive understanding of the mechanisms that regulate ovarian development in animals and new strategies to improve fertility.

## Data Availability Statement

The datasets presented in this study can be found in online repositories. The names of the repository/repositories and accession number(s) can be found below: https://www.ncbi.nlm.nih.gov/, SRR12836353, SRR12836352, SRR12836351, SRR12836356, SRR12836355, and SRR12836354.

## Ethics Statement

The animal study was reviewed and approved by Faculty Animal Care and Use Committee of Northwest A&F University (Shannxi, China).

## Author Contributions

JL and XL conceived and co-ordinated the study. JL performed the study and wrote the manuscript. JL and LG carried out the bioinformatics. XM, YN, CC, SH, and XL gave advices about concept and revised manuscript. All authors read and approved the final manuscript.

## Funding

This study supported by China Agriculture Research System of MOF and MARA (CARS-42) and the Research on the Effect of New Breeding of the Black Muscovy Duck (2019QYPY-130).

## Conflict of Interest

The authors declare that the research was conducted in the absence of any commercial or financial relationships that could be construed as a potential conflict of interest.

## Publisher's Note

All claims expressed in this article are solely those of the authors and do not necessarily represent those of their affiliated organizations, or those of the publisher, the editors and the reviewers. Any product that may be evaluated in this article, or claim that may be made by its manufacturer, is not guaranteed or endorsed by the publisher.
