# Supplementary material for: Integrated ONT Full-Length Transcriptome and Metabolism Reveal the Mechanism Affecting Ovulation in Muscovy Duck (Cairina moschata)
Source: Front Vet Sci. 2022 Jul 8;9:890979. doi: 10.3389/fvets.2022.890979 (PMC9305713; doi:10.3389/fvets.2022.890979)
Supplement: Supplementary file 1 [file Data_Sheet_1.docx]

| **ID** | **Class** | **VIP** | **Fold_Change** | **Log2FC** | **Type** |
| --- | --- | --- | --- | --- | --- |
| MEDP410 | Organic Acid and Its Derivatives | 1.87740262 | 54182.963 | 15.7255517 | up |
| MEDN351 | Lipids | 1.86295015 | 1862.97037 | 10.863389 | up |
| MEDP769 | Benzene and substituted derivatives | 1.86388499 | 1576.54815 | 10.6225535 | up |
| MEDN418 | Carbohydrate metabolomics | 1.50983291 | 1105.53333 | 10.1105268 | up |
| MEDP638 | Lipids_Fatty Acids | 1.71094932 | 130.980865 | 7.03321225 | up |
| MEDN368 | Lipids and Others Phospholipid | 1.86045691 | 51.1854177 | 5.67766095 | up |
| MEDP340 | Lipids and Others Phospholipid | 1.38332521 | 35.8088441 | 5.16224404 | up |
| MEDN366 | Lipids and Others Phospholipid | 1.86578141 | 31.1840896 | 4.96273824 | up |
| MEDP336 | Lipids and Others Phospholipid | 1.81331381 | 26.0477695 | 4.70308793 | up |
| MEDP346 | Lipids and Others Phospholipid | 1.84752007 | 22.4671025 | 4.48974217 | up |
| MEDN362 | Lipids and Others Phospholipid | 1.86862147 | 22.335525 | 4.48126826 | up |
| MEDP494 | Lipids and Others Phospholipid | 1.75415767 | 13.2200866 | 3.72465972 | up |
| MEDP250 | Coenzyme Factor & vitamin | 1.68109851 | 11.7813833 | 3.55843704 | up |
| MEDP344 | Lipids and Others Phospholipid | 1.85723062 | 9.609935 | 3.26452667 | up |
| MEDP352 | Lipids and Others Phospholipid | 1.7761963 | 8.62068512 | 3.10780253 | up |
| MEDN372 | Lipids and Others Phospholipid | 1.81923149 | 7.56629768 | 2.91958754 | up |
| MEDP890 | Others | 1.70315666 | 7.42476046 | 2.89234448 | up |
| MEDP350 | Lipids and Others Phospholipid | 1.80630296 | 6.99169465 | 2.80564218 | up |
| MEDP498 | Lipids and Others Phospholipid | 1.85813825 | 6.8338396 | 2.77269639 | up |
| MEDP338 | Lipids and Others Phospholipid | 1.84736445 | 6.82110549 | 2.77000557 | up |

﻿ **Supplementary Table 1.** Top 20 up-regulated DAMs of ovaries in PO-CO groups.

**Supplementary Table 2.** All the DEGs enriched in the three pathways.

| **ID** | ***P* value** | **log2FC** | **Type** |
| --- | --- | --- | --- |
| gene11743 | 0.00361067 | 2.1000659 | up |
| gene16866 | 0.00475376 | -1.1603879 | down |
| gene1701 | 0.00054865 | -2.0417974 | down |
| gene18741 | 2.37E-10 | -2.9678556 | down |
| gene1992 | 2.03E-05 | -1.8656258 | down |
| gene2244 | 0.0085471 | -3.7520311 | down |
| gene5989 | 5.80E-05 | 2.22355537 | up |
| gene6391 | 0.00012371 | -2.6639755 | down |
| gene7354 | 8.50E-06 | -2.2285731 | down |
| gene7680 | 0.00837654 | -1.1013356 | down |
| gene9070 | 0.00640717 | -1.1600716 | down |
| gene9952 | 0.00144421 | -2.5787518 | down |
| gene9956 | 3.25E-05 | -2.8579971 | down |
| gene9958 | 4.07E-05 | -2.1090482 | down |
| gene12166 | 1.23E-05 | 1.96025325 | up |
| gene12419 | 0.00179882 | 2.20917149 | up |
| gene15484 | 0.00828679 | 0.99724953 | up |
| gene15862 | 0.0002654 | 2.16316076 | up |
| gene16848 | 0.000226 | -1.7667794 | down |
| gene1701 | 0.00054865 | -2.0417974 | down |
| gene17470 | 4.06E-05 | -1.835913 | down |
| gene20796 | 0.00268947 | 1.14031668 | up |
| gene21302 | 0.00095561 | 1.63098453 | up |
| gene6391 | 0.00012371 | -2.6639755 | down |
| gene7826 | 0.00475065 | 2.58220796 | up |
| gene9783 | 0.00025703 | 2.89658263 | up |
| gene9952 | 0.0014442 | -2.5787518 | down |
| gene9956 | 3.25E-05 | -2.8579971 | down |
| gene9958 | 4.07E-05 | -2.1090482 | down |
| ONT.7216 | 0.00280883 | -2.3623282 | down |
| gene11406 | 3.55E-07 | -3.5958654 | down |
| gene12595 | 0.00501541 | -1.0586653 | down |
| gene14404 | 0.00484395 | -2.2836227 | down |
| gene14715 | 0.00312516 | 2.67329547 | up |
| gene14891 | 9.60E-05 | 3.476781 | up |
| gene14942 | 4.11E-07 | -2.1224433 | down |
| gene1794 | 8.39E-10 | 2.88411728 | up |
| gene20701 | 0.00149681 | 2.46757826 | up |
| gene2270 | 3.23E-07 | -2.0541283 | down |

﻿**Supplementary Table 3.** All the DAMs enriched in the three pathways.

| **ID** | **Log2FC** | **VIP** | **Type** |
| --- | --- | --- | --- |
| MEDN352 | -1.2763491 | 1.18378141 | down |
| MEDN372 | 2.91958754 | 1.81923149 | up |
| MEDP336 | 4.70308793 | 1.81331381 | up |
| MEDP338 | 2.77000557 | 1.84736445 | up |
| MEDP340 | 5.16224404 | 1.38332521 | up |
| MEDP342 | 1.77122746 | 1.78832347 | up |
| MEDP344 | 3.26452667 | 1.85723062 | up |
| MEDP346 | 4.48974217 | 1.84752008 | up |
| MEDP350 | 2.80564218 | 1.80630296 | up |
| MEDP352 | 3.10780253 | 1.7761963 | up |
| MEDP434 | 2.74013921 | 1.76328812 | up |
| MEDP494 | 3.72465972 | 1.75415767 | up |
| MEDN536 | 1.0482073 | 1.0527143 | up |
| MEDN793 | -1.6113788 | 1.20274263 | down |
